# Preparation, Modification, Characterization, and Biosensing Application of Nanoporous Gold Using Electrochemical Techniques

**DOI:** 10.3390/nano8030171

**Published:** 2018-03-16

**Authors:** Jay K. Bhattarai, Dharmendra Neupane, Bishal Nepal, Vasilii Mikhaylov, Alexei V. Demchenko, Keith J. Stine

**Affiliations:** Department of Chemistry and Biochemistry, University of Missouri, St. Louis, Saint Louis, MO 63121, USA; jkbxv3@mail.umsl.edu (J.K.B.); dnfzf@mail.umsl.edu (D.N.); bnff8@mail.umsl.edu (B.N.); vmr38@mail.umsl.edu (V.M.); demchenkoa@umsl.edu (A.V.D.)

**Keywords:** nanoporous gold, electrochemical techniques, cyclic voltammetry, amperometry, biosensor

## Abstract

Nanoporous gold (np-Au), because of its high surface area-to-volume ratio, excellent conductivity, chemical inertness, physical stability, biocompatibility, easily tunable pores, and plasmonic properties, has attracted much interested in the field of nanotechnology. It has promising applications in the fields of catalysis, bio/chemical sensing, drug delivery, biomolecules separation and purification, fuel cell development, surface-chemistry-driven actuation, and supercapacitor design. Many chemical and electrochemical procedures are known for the preparation of np-Au. Recently, researchers are focusing on easier and controlled ways to tune the pores and ligaments size of np-Au for its use in different applications. Electrochemical methods have good control over fine-tuning pore and ligament sizes. The np-Au electrodes that are prepared using electrochemical techniques are robust and are easier to handle for their use in electrochemical biosensing. Here, we review different electrochemical strategies for the preparation, post-modification, and characterization of np-Au along with the synergistic use of both electrochemistry and np-Au for applications in biosensing.

## 1. Introduction

Nanoporous gold (np-Au) is a porous three-dimensional (3-D) nanostructure of gold, usually having pore size in the range of a few nanometers to a few hundreds of nanometers [1,2]. This range of pore size includes mesopores (2 to 50 nm) and lower range macropores (51 to around 200 nm) in the International Union of Pure and Applied Chemistry (IUPAC)classification of porous materials [2,3]. The np-Au does not display perfectly circular pores, but mostly consists of open gaps (pores) between the interconnected ligaments [4]. There are many important properties of np-Au, such as high surface-area-to-volume ratio, excellent conductivity, chemical and physical stability, biocompatibility, and plasmonic effects [5,6,7], which intrigue scientists for use in different fields of nanotechnology. Furthermore, np-Au is a suitable surface on which to prepare self-assembled monolayers (SAMs) of functional derivatives of thiolated alkanes, further broadening the interests and applications [8,9]. Np-Au has been explored for application in catalysis [10], optical and electrochemical bio-sensing/assays [11,12], chemical sensing [13], drug delivery [14], carbohydrate synthesis [15], biomolecule separation and purification [16,17], fuel-cell development [18], surface-chemistry-driven actuation [19], and supercapacitor development [20].

Np-Au can be prepared either as a self-supported structure or as a solid-supported structure. Self-supported np-Au is relatively fragile to handle when compared to solid-supported np-Au structures, and it is most commonly used in applications where high total surface area is desired. On the other hand, the solid-supported np-Au structures are physically robust and are mostly used for electrochemical or optical applications. The well-established method to prepare self-supported np-Au is by immersing an alloy of gold and other less noble metals (e.g., Ag, Al, Cu, Zn, Sn, etc.) in concentrated acid or base for different periods of time [1]. Thickness and composition of the alloy, dealloying time and temperature, and concentration of acid or base are the important factors in determining the size of the pores and ligaments [5,6]. For example, submerging the alloy in the concentrated nitric acid for an extended period of time can increase the pore and ligament diameters, due to surface diffusion of atoms causing merging and separation of ligaments [21]. Furthermore, post-annealing using heat can be used for tuning the size of the pores and ligament [5]. The challenges in this field include creating small diameter pores, while completely removing the more reactive unwanted metals, controlled tuning of pores and ligament sizes, and avoiding crack formation. The high surface area of np-Au is desired for electrochemically detecting a small concentration of analytes, however, self-supported np-Au is inconvenient to use as an electrode because of its fragile nature, lack of proper means to handle, and difficulty in separating the area of np-Au from the adjoining metal conductor for the transfer of electrons. Solid-supported np-Au electrodes that are prepared and modified using electrochemical techniques can resolve these problems [4].

Besides np-Au, different other types of nanostructures of gold have been extensively explored and used in various applications [22,23,24]. These gold nanostructures can be grouped based on their dimensions into 0-D (e.g., nanoparticles), one-dimensional (1-D) (e.g., nanorods, nanowires), two-dimensional (2-D) (e.g., nanofilms), and 3-D (e.g., nanocomposite materials, monolithic np-Au) [21,22,23,24]. The 0-D and 1-D gold nanostructures are mainly prepared as a colloidal solution by reduction of gold ions, whereas 2-D nanostructures are surface confined on a solid support using different sputtering or deposition techniques. Zero-dimensional (0-D) and 1-D materials have shown huge potential in clinical settings, mainly for in vivo studies, such as for sensing, imaging, drug delivery, etc. [24]. However, reproducibility of data often becomes challenging in these materials because of chances of agglomeration and necessity of using a stabilizing agent on the surface. On the other hand, 2-D and 3-D gold nanostructures have many applications in a broad range of in vitro studies, ranging from biosensing [22] to organic synthesis [25]. The advantage of np-Au is that it can be prepared in any dimension and shape retaining the properties of nanopores, and hence have wider possibilities than any other form of gold nanostructures. Being such an important and well-explored nanomaterial, there are already many general reviews on np-Au [26,27], but there clearly is a necessity of reviews that are more specific, such as the use of electrochemical methods on different aspects of np-Au.

Here, we review some of the commonly employed electrochemical strategies to prepare, anneal, and characterize solid-supported np-Au electrodes. We further explore the use of np-Au as a transducer in different electrochemical biosensing techniques for detecting chemical or biological molecules. The sensitivity of the electrochemical biosensors depends on the specific surface area and conductivity of the working electrode (transducer). The high surface area allows space for immobilization of a large number of bioreceptors (e.g., single-stranded DNA, enzymes, antibodies, etc.), and hence smaller concentrations of analytes can be detected. Np-Au is highly conductive, as well as having a large specific surface area. Furthermore, the size of its pores and ligaments can be easily tuned in a controlled manner to enable separation of the background matrix from the analyte, which can then enter the np-Au interior, reducing biofouling and increasing sensitivity [28]. On the other hand, both the sensitivity and the selectivity of the biosensor can be improved by proper immobilization of bioreceptors on the surface of the transducer. Np-Au serves as an excellent scaffold for the immobilization of bioreceptors through the physical, chemical, or entrapment methods. Chemical inertness and the physically robust nature of np-Au provide contamination-free surfaces for the immobilization of reproducible amounts of bioreceptors. The ability to form SAMs of functional derivatives of thiolated alkanes and those bearing polyethylene glycol chains on np-Au not only helps bioreceptors to bind strongly on np-Au surfaces, but also to properly orient for analyte binding. Entrapment of enzymes inside np-Au electrodes reduces leaching as well as the tendency for the enzyme to unfold [29,30]. In recent years, many highly sensitive, selective, and stable np-Au-based electrochemical biosensors have been created. In this review, we categorize them based on the type of the bioreceptor used into DNA probe, aptasensor, enzymatic sensor, and immunosensor, and we introduce and discuss different electrochemical techniques and strategies under each category.

## 2. Preparation of np-Au Using Electrochemical Techniques

**Figure 1:**
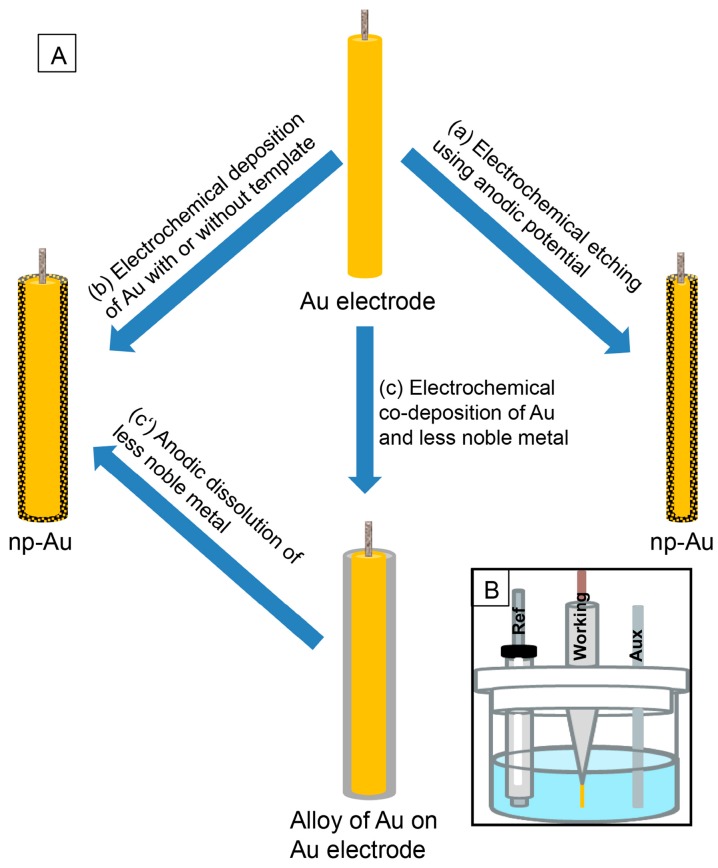
(**A**) Different electrochemical approaches for the preparation of nanoporous gold (np-Au) electrode on gold support: (a) top–down (b) bottom–up, and (c and c′) combination of both bottom–up and top–down approaches. (**B**) Schematic of typical three-electrode electrochemical setup for the preparation of np-Au electrodes.

There are three common electrochemical approaches to prepare np-Au on the solid support. (1) Top-down: by electrochemically etching the pure gold electrode in a suitable electrolyte, (2) bottom-up: by directly electrodepositing gold from solution containing gold ions onto a suitable substrate, and (3) combination of bottom-up and top-down: by selective electrochemical dissolution of less noble metals from the electrochemically co-deposited alloy by applying a suitable anodic potential in different electrolyte solutions (Figure 1A). The typical three-electrode electrochemical setup for preparing np-Au is shown in Figure 1B [21]. It consists of reference, counter, and working electrodes that are submerged in an electrolyte solution and are connected to the potentiostat. Commonly used reference electrodes include silver-silver chloride electrode (Ag/AgCl), mercury/mercurous sulfate electrode (Hg/Hg_2_SO4), and saturated calomel electrode (SCE); platinum (Pt) is the most used counter electrode; and, Au wire, plate, or film can be used as conductive solid support for the preparation of np-Au working electrode.

### 2.1. Etching of Au Electrode

Electrochemical etching of a pure gold electrode is a top-down approach for preparing a supported thin-film of np-Au. The etching of the Au electrode can be performed in situ by the formation of an alloy, oxide film, or a carbonaceous film on the surface of the electrode, which on removal creates a thin layer of np-Au. Multicycle potential scans on a Au electrode in an electrolyte composed of ZnCl_2_ and benzyl alcohol can generate a thin np-Au film at an elevated temperature [31]. Jia et al. were able to prepare a np-Au film by simply cycling the potential 30 times between −0.72 and 1.88 V (vs. Zn) at 120 °C [32]. When the cathodic scan was applied, the alloy of Au–Zn was formed and on the subsequent anodic scan, Zn was removed from the alloy creating a np-Au thin film. The fabrication of np-Au through the formation of a thin-layer of gold oxides and carbonaceous film on the Au electrode can be performed in three steps. The first is to polarize the Au electrode by anodic scanning up to the desired potential at a certain scan rate, the second is to hold the potential for a specific time to generate oxides or a carbonaceous film, and the last is to reverse the potential to regenerate a pure Au surface. Sukeri and coworkers polarized the Au electrode at the scan rate of 0.02 V·s^−1^, held the potential at 2.0 V for 60 min in 0.5 M H_2_SO_4_, and reversed the scan to fabricate a np-Au film with high surface area [33]. The oxide formation on the surface of the electrode was evident by orange-yellowish coloration, which after the electrochemical reduction changed to black confirming the formation of np-Au film. Similarly, scanning and holding the potential at 1.8 V (vs. Hg/Hg_2_SO_4_) in oxalic acid for 90 min [34] and at 4.0 V in citric acid for 3 h [35] can deposit a carbonaceous passivation film on the surface of Au electrode. Removal of this film on the reverse scan can create a np-Au film with uniform pores and ultra-high surface area, with a roughness factor as high as 1000.

Another electrochemical approach to etching the surface of the gold electrode is by dissolving gold atoms in chloride-containing electrolytes. The nanoporous structure of gold evolves in three steps: (1) electrodissolution, (2) disproportion, and (3) deposition [36]. The process starts with the electrodissolution of surface Au atom in chloride-containing electrolyte with the formation of AuCl_2_^−^ and AuCl_4_^−^. This step is followed by the disproportion of AuCl_2_^−^ to Au atoms and AuCl_4_^−^, and finally, deposition of Au atom back onto the gold electrode to create a thin layer of np-Au [36]. Holding a potential as low as 0.9 V (vs. Hg/Hg_2_SO_4_, sat.) in 2 M HCl electrolyte is good enough to generate a np-Au film on a gold electrode. Instead of using acidic HCl, other chloride-containing electrolytes, such as 1 M KCl [13] and 0.5 M NH_4_Cl [37], can also be used to generate the np-Au film at relatively low anodic potential when compared to when non-chloride-containing electrolytes are used.

### 2.2. Electrodeposition of Au

Electrolyte solution containing hydrogen tetrachloroaurate as a source of gold and lead acetate can be used to directly electrodeposit np-Au on a glassy carbon (GC) electrode at low cathodic potential of −0.5 V (vs. Ag/AgCl), increasing the surface area by nearly 16 times [38]. However, a higher cathodic potential of up to −4.0 V versus Ag/AgCl applied for different times was used to prepare np-Au foam from 0.1 M HAuCl_4_ to 1M NH_4_Cl electrolyte on Pt/Ti/Si electrodes [39]. The pore and ligament sizes of the as formed np-Au foam can be further tuned by multi-cycling the potential between 0.4 V and 1.6 V at the scan rate of 50 mV·s^−1^.

### 2.3. Electrochemical Dissolution of Less Noble Metals from Alloy

One of the early works on electrochemical preparation of np-Au was performed using an ionic liquid of zinc chloride-1-ethyl-3-methylimidazolium chloride at 120 °C for both the deposition and dealloying [40]. The gold and zinc binary alloy was electrodeposited on the gold wire, followed by the subsequent removal of less noble zinc from the surface to create np-Au. The pore size and morphology of the nanostructured gold film was tuned by varying the composition of gold and zinc on the surface. However, recent efforts are focused on creating np-Au electrochemically in aqueous medium at room temperature. Commercially available thin alloy films can be electrochemically dealloyed by carefully connecting to the working electrode and applying an anodic potential in a suitable electrolyte to prepare np-Au films. The freestanding np-Au film that is formed by this method is fragile and difficult to handle for future use. To overcome this problem, the alloy can be prepared by electrochemical co-deposition of metal ions on a suitable solid support, followed by dealloying either by submerging in a corrosive medium or by applying an anodic potential.

#### 2.3.1. Alloy Preparation

The electrochemical alloy formation step is an important step in determining the overall surface morphology of the electrode [4]. There are a wide variety of methods for preparing the alloy of gold with less noble sacrificial metals (e.g., Ag, Al, Cu, Zn, Sn, etc.), such as simple chemical co-reduction of metal salts, high temperature melting of different metals, co-sputtering, vapor deposition, and electrochemical co-deposition. For electrochemical co-deposition, Ag is the most preferred metal for gold alloy formation because of its ability to form a homogeneous single-phase face-centered-cubic solid solution across the entire composition range [41]. The alloy is often prepared at the composition ratio of Au_0.2–0.4_Ag_0.6–0.8_ (Au_x_Ag_1-x_) [42]. Phase diagrams of other binary metal-gold alloy and ternary alloy (e.g., Cu–Ag–Au) show difficulty in forming a homogeneous single-phase due to a miscibility gap [26]. Electrochemically deposited Al–Au alloys with 20–50 at. % Au show that phase constitutions of the starting alloys determines the microstructures of the np-Au ribbon [43]. Al-33.4 and 50 Au alloy formed single-phase intermetallic compounds Al_2_Au and AlAu, respectively, whereas Al-20, 30 and 40 Au alloy formed two different phases.

For electrochemical co-deposition, Ag is the most preferred metal and is often prepared as the composition ratio of Au_0.3_Ag_0.7_ (Au_x_Ag_1−x_). The co-deposition rate of both Au and Ag atoms are nearly same because of the same charge, atomic size, and face-centered-cubic structure [41]. This helps to homogeneously distribute and maintain the ratio of gold and silver atoms in the alloy similar to that in the solution.

**Figure 2:**
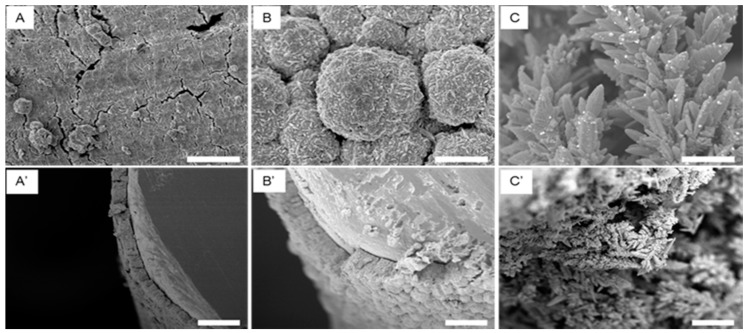
Scanning electron microscopy (SEM) images of nanoporous gold (np-Au)-coated Au wires prepared from 24 h HNO_3_ dealloying of the alloy prepared at (**A**) −1.0 V, (**B**) −1.2 V, and (**C**) −1.4 V for 10 min from 0.015 M KAu(CN)_2_ and 0.035 M KAg(CN)_2_ electrolyte dissolved in 0.25 M Na_2_CO_3_ showing change in morphological features with changing potential. Scale bar: 5 µm. (**A′**), (**B′**) and (**C′**) are the low-magnification cross-sectional of (**A**–**C**), respectively, showing change in thickness with potential. Scale bar: 20 µm. Reproduced from ref. [21] with author’s permission.

**Figure 3:**
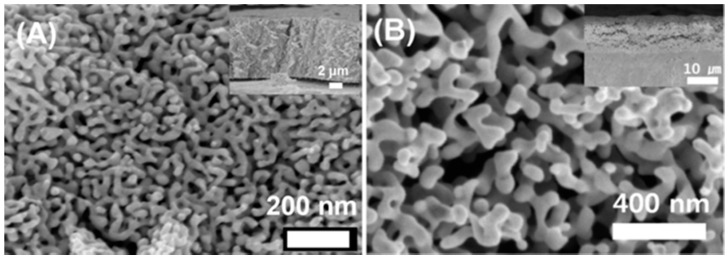
SEM micrographs of nanoporous gold (np-Au) prepared on Au electrode using different strategies, (**A**) Prepared by 24 h dissolution of Ag in concentrated HNO_3_ from Au–Ag alloy prepared by applying potential of −1.0 V (vs. Ag/AgCl) for 10 min on gold wire. Reproduced and slightly modified with permission from ref. [8], Copyright 2016, Elsevier. (**B**) Prepared by anodization with a potential gap of 0.030 V for 300 s in 0.1 M phosphate buffer containing 1 M KCl. Reproduced with permission from ref. [45], Copyright 2014, American Chemical Society. Insets are the SEM images of cross-section of the np-Au showing the boundary between porous and nonporous structure.

Co-deposition potentials of as low as −0.15 V on gold electrode and −0.26 V on glassy carbon (GC) electrode versus Ag wire have been used for the successful deposition of an alloy of Au and Ag from solution of AuCl and AgClO_4_ in 0.1M Na_2_S_2_O_3_ at different molar ratios [44]. The alloy co-deposited at low potential forms a smoother surface when compared to that formed at higher potential where spherical or dendritic structures are formed [21]. Figure 2 shows the low magnification SEM images of np-Au coated Au wire prepared by 24 h HNO_3_ dealloying of the alloy Au_30_Ag_70_ prepared by providing a co-deposition potential of −1.0, −1.2, and −1.4 V for 10 min from 0.015 M KAu(CN)_2_ to 0.035 M KAg(CN)_2_ electrolytes dissolved in 0.25 M Na_2_CO_3_. Clearly, providing −1.0 V forms a smooth structure, −1.2 V forms spherical structures, and −1.4 V forms dendritic structures. The thickness and specific surface area also increase at the more negative potential. Figure 3A,B are the SEM images of np-Au prepared by dealloying the electrochemically prepared Au–Ag alloy in HNO_3_ and by anodizing the Au electrode [45], respectively.

Anodic aluminum oxide (AAO) membranes of different thickness and pore diameter can be used as templates for preparing free or arrays of np-Au nanostructures (e.g., nanorods, nanowires, nanotubes, etc.) [46,47]. Electric contact on one face of AAO is created by sputtering metals, commonly Au for the array and Cu for the free nanostructures, followed by alloy preparation inside the AAO tube by electrochemically co-depositing the metal ions from the electrolyte. In one study, AAO membranes having 100 nm pore diameters were used as templates to prepare Ag–Au alloy from KAu(CN)_2_ and KAg(CN)_2_ electrolyte that was dissolved in 0.25 M Na_2_CO_3_ by applying a potential of −1.2 V (vs. Ag/AgCl) [48]. By dissolving the AAO template and Cu electric contact, alloy nanowires were released and etched in concentrated nitric acid to create np-Au nanowires.

Besides Ag alloys, the electrochemical co-deposition method was also employed for preparing gold alloy or composite with Sn and SiO_2_ in aqueous solution. Commercially available Au–Sn alloy plating solution can be used to electrochemically co-deposit Au and Sn on Ni foam while applying a constant current of 5 mA·cm^−2^ for 5 min at 45 °C [49]. Similarly, Au–SiO_2_ nanocomposite films can be prepared on gold surface by co-electrodepositing Au/Si sol containing different concentration to tetramethoxysilane and KAuCl_4_ applying potential of −0.8 V for 15 min. Finally, Sn and SiO_2_ can be removed from the alloy by chemically etching (1) in 5 M NaOH and 1 M H_2_O_2_ solution at room temperature for three days and (2) in 0.5% and 2.5% HF solution each for 5 min, respectively.

#### 2.3.2. Nano/Micro-Structured Alloy Preparation

Nano- and micro-structures of gold alloy can be prepared in varieties of shapes using co-electrodeposition of metals of interest, which then can be easily dealloyed to np-Au using either the chemical or electrochemical method [50]. These nano- and micro-structures add unique properties and applications to the already useful np-Au, such as creating a superhydrophobic surface [46], which is a substrate for efficiently loading and releasing drugs [51] and sensitive surface-enhanced Raman spectroscopy (SERS) substrates [52,53].

The commonly used method for designing nano- and micro-structured alloy is by using templates. Different types of metal, metal oxides, and metal salts have been employed to design wide varieties of nano- and micro-structures. Anodic aluminum oxide (AAO) membranes of different thickness and pore diameter can be used as templates for preparing free or arrays of np-Au nanostructures (e.g., nanorods, nanowires, nanotubes, etc.) [46,47]. In this method, electric contact on one face of AAO is created by sputtering metals, commonly Au, for the array and Cu for the free nanostructures, followed by alloy preparation inside the AAO tube by electrochemically co-depositing the metal ions from the electrolyte. In one study, AAO membranes having 100 nm pore diameters were used as templates to prepare Ag–Au alloy from KAu(CN)_2_ and KAg(CN)_2_ electrolyte dissolved in 0.25 M Na_2_CO_3_ by applying a potential of −1.2 V (vs. Ag/AgCl) [48]. By dissolving the AAO template and Cu electric contact, alloy nanowires were released and were etched with concentrated nitric acid to create np-Au nanowires. Similarly, Ni-foam can be used to create three-dimensional np-Au film by first electrochemically co-depositing alloy of Au-Sn, followed by removing Sn using NaOH and H_2_O_2_ solution [49]. This method creates np-Au on micrometer size ligaments of Ni-foam. Templates of silver chloride have also been used to prepare wide varieties of nanostructures of np-Au, such as nano-frames, bowls, and shells [54,55,56].

Polystyrene spheres, which can be removed easily using heat or chloroform, can also be used as a sacrificial template to design monolithic hollow spheres [57] and a semi-random array of disks on a silicon or glass surface [53,58]. The size of nano- and micro-structures of alloy can be easily controlled by choosing the appropriate size of the polystyrene sphere.

Dewetting is another suitable method for creating nano- and micro-structures on silica or titanium dioxide surface from layers of gold and less noble metal. Isolated particles or droplets of the alloy are formed by the inter-diffusion of metal layers due to the increase in temperature [59,60,61]. The size and the shape of particles depend on applied temperature, time, and thickness of the metal layers. Once the desired structure of the alloy is formed, it can be easily turned to np-Au using dealloying techniques [62].

#### 2.3.3. Electrochemical Dealloying

The dealloying critical potential for preparing np-Au depends on type, structure, and composition of alloy, as well as the type and concentration of electrolytes and can be determined using linear sweep voltammetry. Diluted acids (e.g., HClO_4_, HNO_3_, and H_2_SO_4_) are commonly used as electrolytes for electrochemical dealloying [63]; however, salts of less noble metals (e.g., AgClO_4_ or AgNO_3_) or their mixture with an acid are also used [44]. The addition of alkali halides, like KCl, KBr, and KI with 0.1 M HClO_4_ as electrolytes have been found to drastically decrease the dealloying critical potential with KI decreasing it by nearly half [64]. The pore size of np-Au was found to be approximately 8 nm without the addition of halides, and changed to 17, 16, and 67 nm with the addition of KCl, KBr, and KI, respectively. The percentage of Au in an alloy is an important factor for electrochemical preparation of np-Au. In an alloy having Au of more than 40 at.%, the dissolution of Ag becomes difficult due to passivation of the surface by the formation of gold oxide, as well as the trapping of Ag inside a higher percentage of Au [65]. The dealloying critical potential is more positive for monocrystalline alloy (111) when compared to polycrystalline alloy having identical composition [66]. The spherical alloy nanoparticles have 0.05 to 0.1 V lower dealloying critical potential when compared to alloy thin films of thickness 20–100 nm, which show comparable dealloying critical potential to the bulk samples [66]. However, very low atomic percentage of gold introduces the frequent appearance of wider cracks due to volume shrinkage [67]. By increasing the atomic percentage of gold from 21.5 to 39 at. % , the cracks on the surface can be drastically decreased [68].

An attempt was made to create np-Au at neutral pH using AgNO_3_ as the electrolyte [69]. Interestingly, the porosity formation occurred only above 1.3 V (vs. NHE) in the region where the Pourbaix diagram suggested that the passivation of the surface occurs due to silver oxide formation. The reason for the pore formation was explained using pitting and crevice corrosion. The authors hypothesized that due to the Ag and water oxidation at higher potential, the proton gradient increases at the dissolution front falling into the corrosion region of the Pourbaix diagram dissolving the silver and creating np-Au. A pore size as small as 5 nm can be prepared using this method; however, a significant amount of residual Ag may be left, which can be removed by removing the oxides layers by treatment with 0.025 M Na_2_SO_4_ at 0.24 V. A larger pore size can be created by removing the oxides layer in between the dealloying process. In another study, 10 wt. % NaCl was used as an electrolyte to prepare np-Au ribbon form the Al–Au alloys with 20–50 at. % Au under different potentials [70]. Pourbaix diagram and chloride ion effect were used to explain the dealloying mechanism in neutral NaCl solution. It was found that with the increase in potential form 1.5 to 2.0 V, the ligament size and frequency of micro-cracks increase remarkably. Table 1 summarizes and compares the advantages and disadvantages of different np-Au fabrication techniques.

## 3. Post-Annealing of np-Au

The size of pores and ligaments of np-Au can be tuned in situ while creating np-Au just by varying the preparation conditions. However, when desired or required, post-annealing of np-Au can be performed to tune the size of pores and ligaments. Thermal annealing is the commonly used technique for the post-annealing process [71]. By annealing np-Au at temperatures above 300 °C for 10 min or more, the number of pores in a specific area can be drastically decreased [72]. This is because of the formation of micro-cracks due to merging of adjacent nanopores while increasing the width of ligament nodes. It can also be because of contraction of the sample (volume shrinkage) due to an increased temperature. However, suitable temperature and time in the thermal annealing process depends on the thickness and nature of the sample [72]. The large cracks that are formed during the dealloying process can be reduced by annealing at around 300 °C [67]. Photothermal annealing of np-Au can be performed using either a continuous-wave laser or a pulsed laser mill. The average pore and ligament size of np-Au can be increased by increasing the intensity of the laser [73]. Photothermal annealing using a laser is not only useful in tuning the pore and ligament sizes, but also in creating a library of np-Au having a wide range of morphologies on a single chip [74]. Recently, a novel electro-annealing method was developed for a precise control of np-Au morphology at low temperature [75,76]. This method anneals np-Au by applying a constant current at low temperature (<150 °C), assisting the thermally activated surface diffusion of Au atoms, and hence, coarsening.

**Figure 4:**
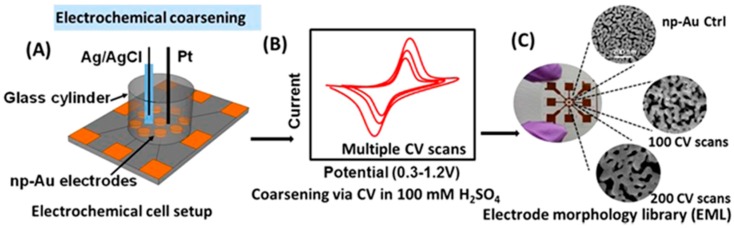
Electrochemical coarsening (**A**) Integrating nanoporous gold (np-Au) multiple electrode array into the electrochemical cell and (**B**) cycling each electrode in H_2_SO_4_ using cyclic voltammetry (CV) to (**C**) create an electrode morphology library having diverse coarsened morphologies on a single chip. Reproduced with permission from ref. [7], Copyright 2017, American Chemical Society.

The advantage of electrochemical annealing of np-Au is that it can be performed quickly at room temperature while increasing the pore size without increasing the ligament width [77]. In one reported method, the potential pulses of varied width and duration were applied between 0 and 1.1 V (vs. Pt) in a two electrode cell containing aqueous HCl. Application of the 1.1 V potential pulse resulted in the formation of chloroaurate (AuCl_2_^−^) complexes that diffused away from the gold surfaces, resulting in uniform ligament thinning. Our lab has explored the effects of three different electrolytes NaNO_3_, NaClO_4_, and KCl on np-Au annealing using potential cycling [4]. This was done by providing oxidation-reduction cycles between −0.4 V to +1.2 V versus Ag/AgCl reference electrode at scan rate of 100 mV·s^−1^ using a 2 s hold at the positive end and 8 s hold at the negative end. SEM images showed that larger but fewer pores are found after the annealing without thickening the ligaments in all three electrolytes. However, the surface area of np-Au (21 ± 2 cm^2^) is drastically decreased when annealed in KCl solution to 7 ± 1 cm^2^ after the 30 cycles when compared to NaNO_3_ and NaClO_4_ annealed samples with 16 ± 1 and 16 ± 2 cm^2^, respectively. The decrease in surface area in KCl was attributed to formation of chloroaurate species, which was confirmed by absorbance spectroscopy to be present in the solution after potential cycling. Electrochemical annealing is also a suitable method to remove any residual silver from np-Au structure that otherwise would not be possible using thermal annealing. Matharu and co-workers demonstrated that the size of interligament gap and ligament width can also be increased by multiple CV scans of the np-Au electrode over the potential range of 0.3–1.2 V in H_2_SO_4_ [7]. The size of interligament gap and ligament width nearly doubled after 100 CV scans (Figure 4).

## 4. Self-Supported np-Au Electrode

The self-supported np-Au prepared using wet chemical methods can be used as an electrode for biosensing; however, there are some challenges. Once completely dealloyed using nitric acid, np-Au is fragile to handle and modify. It is also challenging to separate the boundary between np-Au and the adjoining conducting surface of the working electrode. Despite these facts, many studies have been performed where chemically prepared np-Au films have been successfully immobilized on different types of solid supports to be used as a working electrode [78]. An intermediate layer of dithiols (e.g., hexanedithiols) can be used to increase the adhesion between chemically prepared np-Au film and planar gold electrode surface [79]. Stable and conductive polymers, like Nafion, can be used to stabilize the np-Au films on glassy carbon [80] or indium tin oxide (ITO) surface [81]. In many studies, dealloyed gold leaf was transferred onto the surface of a glassy carbon electrode to create a supported np-Au electrode.

## 5. Electrochemical Characterization of np-Au

The surface morphology along with the size of pores and ligaments of np-Au is commonly characterized by scanning electron microscopy (SEM). Transmission electron microscopy (TEM) is useful in analyzing thin np-Au films at better resolution. Energy dispersive X-ray spectroscopy (EDS) provides information about the elemental composition, which helps to confirm the removal of sacrificial metals from np-Au.

**Figure 5:**
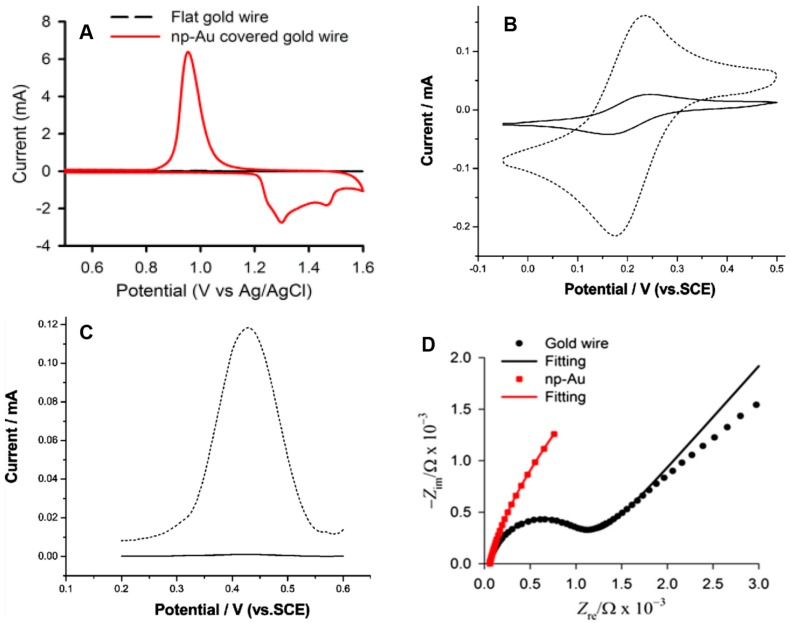
Comparison of different electrochemical behaviors of flat Au versus nanoporous gold (np-Au) electrode. (**A**) Cyclic voltammetry (CV) used to determine the surface area of the electrodes usingthe oxide stripping method, obtained in 0.5 M H_2_SO_4_ at a scan rate of 100 mV·s^−1^ (vs. Ag/AgCl). (**B**) CV acquired using 10 mM Fe(CN)_6_^3−/4−^ in 0.1 M KCl solution at scan rate 50 mV·s^−1^ (vs. saturated calomel electrode (SCE)) to test the kinetics of the electrode interface. (**C**) square-wave voltammetry curves of flat gold electrode (solid line) and np-Au (dotted line) acquired using 10 mM NH_4_FeSO_4_ in 0.1 M KCl solution obtained under a frequency of 50 Hz, increments of 4 mV, and amplitude of 25 mV. (**D**) Nyquist plots of electrodes obtained using 10 mM PBS at pH 7.4 containing 5 mM K_3_[Fe(CN)_6_] and 5 mM K_4_[Fe(CN)_6_] redox probe. (**B**,**C**) Reproduced with permission from ref. [32], Copyright 2007, American Chemical Society. (**A**,**D**) Reproduced with permission form ref. [8], Copyright 2016, Elsevier.

On the other hand, electrochemical techniques are useful in understanding the kinetics at the np-Au interface and to determine the surface area. Cyclic voltammetry (CV) is the method of choice for determining the surface area of np-Au electrode. In CV, the potential of the working electrode is cycled at a particular scan rate, creating a triangular potential waveform and measuring the resulting current. Figure 5A shows the CV that is used for the determination of microscopic surface area of np-Au by oxide stripping method. In this method, an anodic scan is applied on a clean np-Au electrode immersed in diluted H_2_SO_4_ to form a gold oxide layer, which is then reduced on the subsequent reverse scan. The charge under the cathodic peak is measured, and the surface area of np-Au is estimated using the known amount of charge required to reduce gold oxide from a square centimeter of the gold surface. However, there is always the chance of uncertainty in this value because of the double-layer charging, contribution from other Faradaic processes, and variation of the crystal face of the metal [82]. The two most commonly reported values are 386 µC·cm^−2^ [82] and 450 µC·cm^−2^ [83]. Figure 5A is a comparison between cyclic voltammograms of flat gold wire (black) and np-Au wire (red), clearly showing a substantially higher surface area of np-Au wire. The roughness factor (Rf) of the np-Au against the planar surface can be obtained by dividing the microscopic surface area by the geometric surface area, which can range from tens to thousands depending on preparation methods, amount of material, and the size of the pores [32,35].

CV and square wave voltammetry (SWV) can be used for determining the electrochemically active area of np-Au. In Figure 5B,C, the solid lines represent the kinetic response of the redox probes on planar gold, and the dotted lines are on np-Au, clearly showing the enhancement of the response [32]. Electrochemical impedance spectroscopy (EIS) is used for measuring the resistivity of the np-Au electrode. A clean flat gold wire always produces a small characteristic semicircle (represents the charge or electron transfer resistance to and from the electrode surface) and a straight line (represents Warburg impedance due to diffusion), Figure 5D. A straight line can also be observed for np-Au electrode, but it lacks the semicircle, and it is a characteristic Nyquist plot of a porous structure [8].

## 6. Electrochemical Biosensing

The range of pore sizes that are typically found for np-Au, in the range of 20–100 nm, make the pores suitable for immobilization of a variety of biomolecules, while retaining access for their binding partners or substrates. The classes of biomolecules that have been immobilized on np-Au for development of electrochemical biosensors include antibodies, aptamers, single-stranded or double-stranded DNA, enzymes, lectins, and other proteins. Analytes detected by electrochemical biosensing on np-Au include single-stranded DNA, small molecules, metal ions, and various proteins. Unmodified np-Au has also been used for enhanced electrochemical sensing; for example, phenol and catechol can be detected with high sensitivity on a np-Au thin film electrode by applying a suitable potential [84,85]. As another example, a np-Au electrode has been used to detect arsenite ion [As(III)] to a concentration as low as 0.0315 ppb with the sensitivity of 44.64 µA·cm^−2^·mM^−1^ [86]. We will focus our discussion on the electrochemical detection of analytes on np-Au by dividing biosensors that are based on the type of immobilized receptor: DNA (both aptamers and hybridization-based sensors), enzymatic sensors, and immunosensors. We include studies that were carried out on np-Au prepared by dealloying, as well as by other methods.

### 6.1. DNA Sensor

#### 6.1.1. Aptamer-Based Electrochemical Sensors

Aptamers are single strands of DNA that fold into a three-dimensional structure, which results in their binding with high specificity to a chosen target molecule. The optimal aptamer for a given target molecule is generated by the combinatorial evolutionary process, known as systematic evolution of ligands by exponential enrichment (SELEX) [87]. Aptamers have advantages that include their stability, ease of re-use, broad range of possible targets, synthesis using well-established oligonucleotide technology, and relatively small size [88]. Aptamers can be synthesized with a thiolated end for direct assembly onto np-Au or Au. In one study, a 5′-thiolated form of an aptamer binding to postoperative lung cancer tissue and related circulating tumor cells was assembled onto a gold electrode and then backfilled with mercaptoethanol. Clinical blood plasma samples from healthy and lung cancer patients were studied. The binding of the cancer-related proteins to the aptamers on the gold electrode resulted in a reduction of the peak current due to the ferrocyanide/ferricyanide redox couple, as detected by square-wave voltammetry [89]. The signal was enhanced by the use of hydrophobic beads binding to the proteins bound to the aptamers. The samples from the cancer patients inhibited the peak current significant more than samples from healthy patients. In another study, aptamers against cardiac troponin I (cTnI), which is a biomarker for acute myocardial infarction, were developed using SELEX and then immobilized on Au in their 5′-thiolated form. The electrode surface was then filled in with mercaptohexanol to limit nonspecific binding. The binding of cTnI to the aptamers reduced the peak current from the oxidation of ferrocene on ferrocene-modified silica nanoparticles, as detected by square-wave voltammetry.

Aptamers have been immobilized on np-Au in a small number of studies thus far. An aptamer for the protein thrombin was immobilized onto np-Au prepared by applying a square-wave pulse to a gold electrode [90]. The thiolated aptamer was immobilized on np-Au so that it could bind to one binding site on thrombin. Subsequently, after binding of thrombin, Au nanoparticles decorated with aptamer and non-binding ssDNA were allowed to bind to another binding site on the thrombin. Detection was achieved by chronocoulometry oxidation of [Ru(NH_3_)_6_]^3+^ electrostatically bound to anionic phosphodiester bonds of the oligonucleotide backbone with the amount bound proportional to the amount of bound ssDNA which was proportional to the amount of bound thrombin. The aptasensor had a linear range from 0.01 to 22 nM and could detect thrombin to as low as 30 fM with good selectivity. In another study, an aptamer for adenosine triphosphate (ATP) was split into two fragments prior to immobilization [91]. The first thiolated fragment was immobilized on np-Au that had been prepared by anodization, followed by reduction by ascorbic acid. After binding of ATP, the second fragment of the aptamer was bound and then conjugated to the redox marker 3, 4-diaminobenzoic acid, which was detected by differential pulse voltammetry. The response, although nonlinear, covered a wide range of ATP concentration from 0.1 to 3000 µM. An aptamer against bisphenol A was immobilized on np-Au electrode that was prepared by affixing dealloyed gold leaf onto a glassy carbon electrode [92]. Filling in the surface with mercaptopropionic acid was used to limit nonspecific adsorption. The steps of the surface modification were characterized using electrochemical impedance spectroscopy. In the case of this aptasensor, the bound bisphenol A was electroactive and was detected by differential pulse voltammetry. Selectivity against molecules of similar structure, such as bisphenol B, was demonstrated.

In addition to the synthesis of aptamers, it is also possible to create ssDNA with catalytic activity, referred to as a DNAzyme [93]. A Pb^2+^ dependent DNAzyme was immobilized onto np-Au that was prepared from dealloyed foils affixed onto glassy carbon. A capture probe ssDNA was immobilized onto np-Au, followed by binding of Au nanoparticles modified by a partially complementary ssDNA. Addition of Pb^2+^ activated the capture probe ssDNA to give DNAzyme activity whereby cleavage of the complementary strand was achieved. Detection was achieved by chronocoulometry after exposure of the electrode to [Ru(NH_3_)_6_]^3+^. An increase in Pb^2+^ resulted in enhanced DNAzyme activity, cleavage of the complementary strands, and hence, binding of less [Ru(NH_3_)_6_]^3+^ and a lower total charge detected by chronocoulometry.

#### 6.1.2. DNA Hybridization-Based Electrochemical Sensors

The hybridization of ssDNA immobilized on np-Au with a complementary strand has also been used as the basis for the development of electrochemical biosensors. Many of these biosensors have used for detection of complementary ssDNA strands, and the hybridization events are detected by the electrochemical signal from electroactive bound or covalently attached redox probes. For example, a thiolated ssDNA capture probe can be assembled onto a gold electrode such that an outer partial length of the capture probe will hybridize with a portion of the target DNA strand [94,95]. The reporter DNA strands, which are bound to Au nanoparticles, will then bind to the remainder of the target probe length. Signal amplification was achieved by the numerous reporter strands on the Au nanoparticles. Exposure of this sandwich-like assembly to [Ru(NH_3_)_6_]^3+^ resulted in the binding of [Ru(NH_3_)_6_]^3+^ by electrostatic interaction with the negatively charged phosphates of the oligonucleotides. Chronocoloumetry was used to determine a total charge that was associated with bound [Ru(NH_3_)_6_]^3+^, which was proportional to the amount of bound DNA. The method was able to detect target DNA down to femtomolar levels. DNA that was associated with the breast cancer gene BRCA1 was used as a target. Signals due to DNA with single oligonucleotide mismatches were less than 20% those for the exact complementary sequences. In the preparation of these modified electrodes, the ssDNA capture probe assembled on the Au surface is subject to additional assembly of a molecule, such as mercaptohexanol, which makes the DNA strands stand up better and inhibits nonspecific interactions. The electrostatic binding of [Ru(NH_3_)_6_]^3+^ to ssDNA on Au, as assessed by chronocoulometry, has been used to determine the surface density of ssDNA in mixed monolayers with mercaptohexanol on Au [96]. The method was also used to determine surface coverage of dsDNA, and hence the hybridization efficiency as a function of surface coverage of ssDNA. Hybridization was found to be efficient up to a threshold probe surface density value.

**Figure 6:**
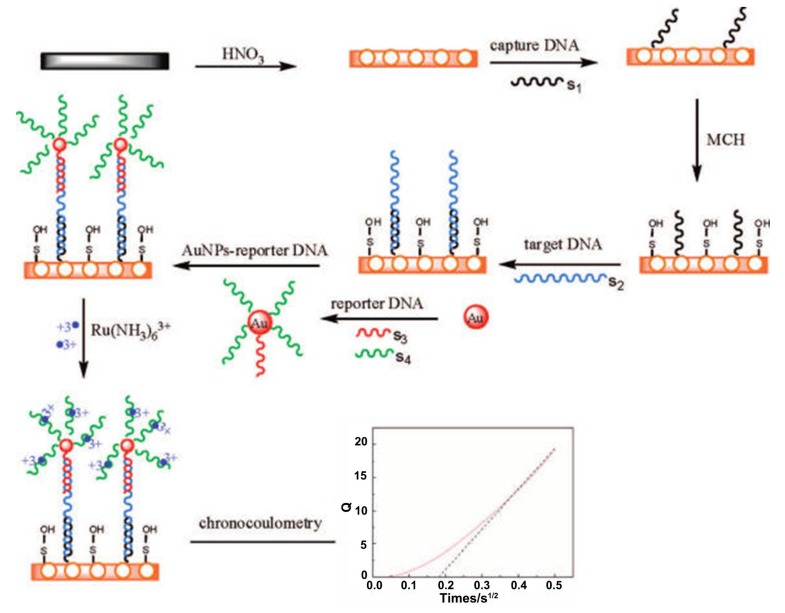
Scheme of chronocoulometry determination of DNA hybridization through two steps of amplification. Reproduced with permission from ref. [98], Copyright 2008, American Chemical Society.

A np-Au electrode prepared using square-wave oxidation reduction cycling (SWORC) was used to create a hybridization-based sensor for DNA of the gene for the surviving protein that is associated with osteosarcoma [97]. The capture probe DNA was immobilized and then oriented and backfilled with mercaptohexanol. The hybridization of the target DNA was monitored by chronocoulometric detection of the additional bound [Ru(NH_3_)_6_]^3+^ to the hybridized structure. Detection of the target DNA down to 5.6 fM was possible, and was selective against single base mismatches. In another study, a np-Au electrode was modified with the DNA capture probe [98]. Au nanoparticles were prepared being modified with both reporter DNA complementary to the target strand and also DNA not complementary to the target strand whose purpose was to reduce cross-reaction between the target and reporter. Detection of DNA binding was done by chronocoulometric detection of [Ru(NH_3_)_6_]^3+^ electrostatically bound, and a detection limit of 28 aM was achieved with selectivity against single base pair mismatches. The scheme for this analysis is reproduced in Figure 6.

In another approach, ferrocene modified DNA probes were used to develop a sensor based on hybridization on a np-Au electrode [99]. Ferrocene carboxylic acid was conjugated to the amino-labeled DNA probe using EDC/NHS coupling chemistry. In this study, differential pulse voltammetry was used to measure the current after exposure of the immobilized capture probe to target DNA that was complementary, singly mismatched, or non-complementary, and then to the ferrocene labeled reporter DNA strand. The sensor, as designed, had good selectivity and could detect as little as 25 pmol of target DNA. A sensor for the genomic DNA of *E. coli* was developed on np-Au electrodes using methylene blue as a redox probe, as it binds with higher affinity to ssDNA than to dsDNA [100]. Using differential pulse voltammetry (DPV), successively lower peak currents from methylene blue were found upon exposure of the capture probe modified DNA with genomic DNA from greater amounts of colony forming units (CFU) per µL of *E. coli*. The decrease in DPV peak current due to decreased methylene blue binding to hybridized DNA was also used to create a sensor on np-Au for the PML/RAR fusion gene associated with acute promyelocytic leukemia [101]. In this case, the np-Au electrode was created using SWORC. A detection limit of 6.7 pM target DNA was achieved.

Stripping voltammetry has also been used to create a hybridization-based sensor on np-Au [102]. In this study, Au nanoparticles were labeled with both reporter DNA strands, and with DNA strands that were linked to lead sulfide (PbS) nanoparticles. After target hybridization to the immobilized capture probe DNA and binding of the reporter probe modified nanoparticles, the assemblies were dissolved using nitric acid. The lead ion content was detected by differential pulse anodic stripping voltammetry. A detection limit of 0.26 fM was achieved with good selectivity.

The interaction of Hg^2+^ with stem-loop DNA probes immobilized on np-Au was used to create an electrochemical hybridization based sensor for Hg^2+^ [78]. Hg^2+^ can bind between two thymine bases and promote the formation of stable thymine–Hg^2+^–thymine base pairs and the opening of the stem-loop capture probe. The binding of a complementary strand, labeled with ferrocene, resulted in a peak current in DPV that increased with Hg^2+^ concentration. A detection limit of 0.0036 nM was achieved with excellent selectivity against other dissolved metal ions.

The Seker lab has explored the effects of np-Au morphology and pore size on DNA hybridization sensing [7,11,16]. The decreased binding of methylene blue upon hybridization was detected by square-wave voltammetry [7]. It was found that there were different optimal square-wave frequencies for maximizing the current response on np-Au as prepared and thermally annealed. The hybridization could be detected to as low as 500 pM on annealed np-Au, which was a 10× lower detection limit than on np-Au, as prepared. Np-Au, with pore size that as comparable to that of proteins such as bovine serum albumin or those in fetal bovine serum used as a simulant of human serum, was found to resist biofouling when used as an electrochemical sensor for DNA hybridization [11]. The np-Au with pore size 14 nm was shown to show a minimal effect of concentrations of bovine serum albumin of 2 mg·mL^−1^ on the response of methylene blue redox probe to DNA hybridization (26 bp), as assessed by square-wave voltammetry. The capture of target DNA from fetal bovine serum by np-Au modified with capture probe DNA was followed by the release of the hybridized DNA by reductive desorption, carried out by cyclic voltammetry scans between 0 and −1.5 V (vs. Ag/AgCl) [16]. Creation of a library of np-Au morphologies on a chip was used to optimize the performance of the DNA hybridization sensors [7]. Table 2 summarizes the analytical response characteristics, and the type of recognition, either aptamer or hybridization, for the DNA based sensors that are discussed in these two sections.

Besides large active surface area, np-Au with the optimal size and geometry was found to act as sieves, allowing for only targeted analyte and redox probe inside np-Au, while blocking the transportation of the complex media. This process highly reduces the biofouling and nonspecific interactions with the receptors [28]. Seker’s group used SWV to detect target DNA molecules using capture probe immobilized on np-Au surface [28]. They were able to detect 10 nM to 200 nM DNA molecules in physically relevant complex media. Unlike for chronocoulometry (CC) and DPV, the tagging/labeling step is unnecessary in SWV based on np-Au. The group has shown that the electrochemical current response during probe and target DNA hybridization can be amplified 10-fold using np-Au when compared to planar Au electrodes [11]. In a recent work by this group, multiple np-Au electrode arrays having different morphology were microfabricated on a single chip to study DNA hybridization [7]. Using SWV, it was concluded that np-Au, with a range of average pore radii of 25–30 nm, facilitates the permeation of target DNA, while inhibiting the nonspecific adsorption of the background proteins. The comparison among some of the electrochemical techniques in terms of their sensitivity for the detection of varieties of analytes is shown (Table 1). However, there are other electrochemical techniques, such as EIS and CV, which frequently are used as complementary tools along with CC, DPV, and SWV for electrochemical aptasensing [91,97].

### 6.2. Enzymatic Sensor

Enzyme-based electrochemical biosensors are designed based on the way that they transfer electrons to the surface of the electrode. The two common ways of transferring electrons are through mediated electron transfer (MET) and direct electron transfer (DET) [103]. In the MET-based biosensor, a suitable mediator is either added to the solution or polymerized or co-immobilized along with the redox enzymes [104]. This method can rapidly transfer electrons to the electrode, but has several drawbacks that are associated with mediators, such as leaking, non-selectivity, diffusion limitations, short lifespans, and poor biocompatibility [104]. DET can avoid some of these problems by directly transferring electrons from the redox active center to the surface of the electrode. However, the redox active center of some oxidoreductases is deeply buried inside the insulating protein shell, thus hindering the DET process [105]. Proper orientation of the enzyme and minimal distance from the active centers to the surface of the electrode can highly enhance the DET. Np-Au is found to be a suitable support for DET of enzyme-based electrochemical sensors [106,107,108,109]. This is due to (1) biocompatible nature of np-Au to maintain the enzymatic activity of an enzyme [1], (2) constraint of porous environment to increase stability of enzyme by decreasing the tendency to unfold [29,30], (3) tunability of pore and ligament sizes to reduce leaching of trapped enzymes [29], (4) SAMs forming ability to strongly bind enzymes with an increase in loading and decrease in leaching [110,111], and (5) high surface area to amplify the response of the analyte [112]. Enzymes, such as horseradish peroxidase (HRP) [106], human cytochrome P450 [107], laccase [108], and bilirubin oxidase [109] have already been immobilized on np-Au with an enhancement in the DET process. The np-Au immobilized enzymes are also useful in biofuel cells, where np-Au anode and cathode are modified with two different enzymes to generate high power density [104,113]. Chronoamperometry (CA), or amperometry, is one of the most widely used electrochemical techniques for enzyme-based biosensing where current is monitored with the applied potential over time. However, other electrochemical techniques, such as CV and DPV, can also be used.

#### 6.2.1. Glucose as an Analyte

Sensitive and reliable methods for the detection of glucose concentration in human blood are important for the identification and management of diabetes. According to American Diabetic Association, a fasting plasma glucose level below 5.55 mM is considered to be normal, 5.55–6.94 mM is considered prediabetic, and equal or above 6.99 mM is considered diabetic. An ideal glucose detector should not only be selective and sensitive, but also be inexpensive and easy to use with a short processing time.

Np-Au has shown promising results in glucose detection without further modifying it with any enzyme. The response is due to its highly selective activity towards glucose, while avoiding interference from the commonly available interferents in the bodily fluids, such as ascorbic acid (AA), uric acid (UA), and chloride ions [114]. The enzyme-free np-Au-based glucose sensors are not only stable and simple to prepare, but also improve the reproducibility with the possibility of reusing the test stripes [115,116]. The final product of the non-enzymatic electrochemical oxidation of glucose is gluconic acid through the two-electron oxidation process. The α- and β-glucose first oxidized to gluconolactone, followed by hydrolysis into gluconic acid, whereas γ-glucose (the linear free aldehyde form) is directly oxidized into gluconic acid [116]. In general, non-enzymatic electrocatalysis of glucose occurs via its weak adsorption on the electrode surface where it undergoes breaking and formation of new bonds [116]. Two glucose oxidation peaks at around −1.0 V and 0.25 V (vs. Ag/AgCl) can be observed when scanned in 0.1 M phosphate buffer (pH 7) [117]. The presence of chloride ions in the electrolyte solution was found to only interfere with the signal at −1.0 V, but the oxidation peak at 0.2 V is not much affected [117]. In fact, the sensitivity of np-Au having very high surface area (Rf around 1000) can be greatly enhanced by the addition of chloride ions [35]. It is believed that the fast-moving chloride ions may help the slow glucose oxidation reaction to utilize the entire np-Au surface, which otherwise would not have been possible. Along with the surface area, the pore size of np-Au and pH of the electrolyte are important factors in determining the sensitivity of the electrode. The np-Au, having a pore size of 18 nm, gives better response when compared to np-Au with 30, 40, and 50 nm pores, whose sensitivity are in decreasing order [118]. Np-Au shows better sensitivity when the electrolyte is alkaline (e.g., NaOH and KOH) when compared to physiological pH 7.4 (e.g., PBS buffer). Zhou and co-workers have determined that sensitivity as high as 3625 µA·cm^−2^·mM^−1^ can be achieved when np-Au having moderate Rf (215) at 0.2 V (vs. SCE) is used for the glucose detection in NaOH solution. The same electrodes in PBS have a sensitivity of 307 µA·cm^−2^·mM^−1^ at −0.1 V (vs. SCE), and in the PBS containing chloride ions have only 73 µA·cm^−2^·mM^−1^ at 0.2 V (vs. SCE).

Although the enzyme-free np-Au show promising advantages for the selective and sensitive detection of glucose, most of the commercially available glucose detectors are still based on glucose oxidase (GOx) [126]. Enzymatic glucose sensors are classified into three generations that are based on the electron transfer mechanism. The first generation uses dissolved oxygen and amperometric detection of the H_2_O_2_ byproduct, the second generation uses mediators (small organic or inorganic molecules), and the third generation uses direct oxidation by the electrode to catalytically oxidize the reduced flavin adenine dinucleotide (FADH_2_) to flavin adenine dinucleotide (FAD) in the GOx [127]. The FADH_2_ is formed by the initial reduction of FAD due to the oxidation of glucose into gluconic acid. Np-Au has been successfully utilized to physically or covalently immobilize GOx on its surface and detect the glucose concentration (Table 3). Covalent immobilization can be achieved by forming self-assembled monolayers of different organic molecules having terminal functional groups through which GOx can be captured on the surface of np-Au. Loading the GOx inside the np-Au using physical interactions might be less effective due to the leakage of enzymes from np-Au with larger pore sizes. This problem has been addressed using Nafion, which not only protects against leakage of the enzymes from the pores, but also reduces the influx of glucose inside the np-Au [30,120] and minimizes the interference of ascorbic acid (AA) and proteins in biological samples [35]. GOx-based np-Au sensors can be prepared based on dissolved oxygen [110] or using suitable mediators, such as Prussian blue [120], p-benzoquinone [122], ferrocenecarboxylic acid [122], and osmium containing polymers [121]. The mediators can be dissolved in the electrolyte or immobilized on the np-Au surface along with the GOx. The Prussian blue and GOx modified np-Au electrode can have sensitivity of 50 µA·cm^−2^·mM^−1^, with a wide linear range of 2–30 mM and the limit of detection (LOD) of 300 µA at 0 V (vs. Ag/AgCl). However, changing the potential to −1.0 V (vs. Ag/AgCl) can drastically decrease the LOD to 2.5 µA [119,120]. Although GOx-based np-Au glucose sensor showed linear response in the clinically desired concentration range and provided good selectivity and sensitivity, there are other challenges that are associated with this sensor. GOx is relatively stable at room temperature when compared to many other enzymes; however, denaturation or loss of activity may occur due to long storage time, heat, and chemicals [128]. Furthermore, the loss of response may occur due to leakage of enzymes from np-Au [30], and the electrode preparation steps can be time-consuming and tedious [128].

The sensitivity and selectivity of np-Au-based glucose sensor can also be enhanced by modifying it with different metals (e.g., Ni, Cu, Pt, Ru, and Pd) [129,130,131,132,133] metal oxides (e.g., CuO, Ni(OH)_2_ and Co_3_O_4_) [134,135,136] and alloys (e.g., PtCo) [137]. As-modified np-Au can also be used for the detection of varieties of other analytes, such as L-cysteine, by molecularly imprinted polymer and Cu NPs that are immobilized on np-Au [38], hydrogen peroxide and hydrazine by np-Au prepared on Ni foam [138], and Cr(VI) by depositing Ag NPs on np-Au [139].

#### 6.2.2. Other Small Molecules as Analyte

**Figure 7:**
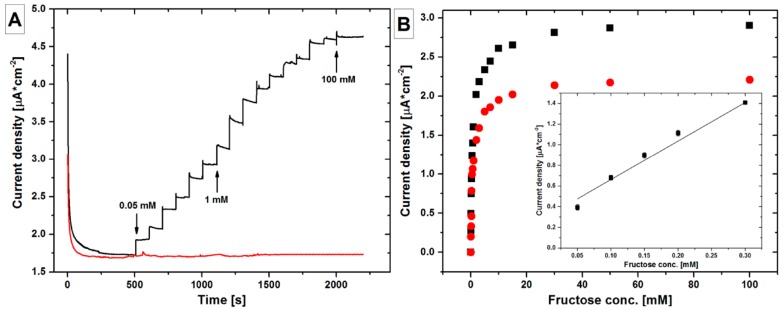
(**A**) Plot of current density obtained at, 2-carboxy-6-naphtoyl diazonium-fructose dehydrogenase (ND-FDH) modified np-Au electrodes as a function of fructose concentration. The arrows indicate the fructose concentration at different times. (**B**) Calibration plots obtained at 18 (●) and 42 nm (■) pore size electrodes. The inset shows the linear range of the biosensor (25 °C at pH 5.5). Conditions: applied potential of 0.15 V. Reproduced with permission from ref. [140], Copyright 2017, Wiley-VCH Verlag GmbH & Co. KGaA, Weinheim.

One of the recent works involves the immobilization of fructose dehydrogenase on a np-Au surface for sensitive and selective detection of d-fructose using chronoamperometry (Figure 7) [140]. The enzyme fructose dehydrogenase orients on np-Au surface exposing its FAD and heme subunits. The FAD subunit catalytically converts d-fructose to 5-dehydro-d-fructose, while reducing itself to FADH_2_. The reduced form can then be oxidized back transferring the electron to heme subunit, which in turn, can then directly transfer electron at electrode surface. The biosensor prepared using this method was able to detect fructose in the linear concentration range from 0.05 to 0.3 mM, with the response time of less than 5 s and Michaelis-Menten constant (K_m_^app^) of 0.68 ± 0.04 mM. The detector has the limit of detection 1.2 µM with the sensitivity of 3.7 ± 0.2 µA·cm^−2^·mM^−1^. Enzyme-based electrochemical sensors are also an excellent way to detect or catalyze a small concentration of hydrogen peroxide. Lu et al. modified the np-Au surface with horseradish peroxidase to detect H_2_O_2_ using CA [106]. The apparent electron transfer rate constant was found to be 2.04 ± 0.12 s^−1^. The biosensor was able to detect H_2_O_2_ at a low overpotential, with a sensitivity of 21 μA·mM^−1^. The linear range of the biosensor was 10–380 μM, with a detection limit of 2.6 μM. Enzyme-based np-Au biosensors are also used to detect lipids, such as cholesterol and triglycerides. Ahmadalinezhad and Chen co-immobilized three different enzymes, cholesterol oxidase (ChOx), cholesterol esterase (ChE), and horseradish peroxidase (HRP), on np-Au surface prepared on titanium surface (Ti/NPAu/ChOx–HRP–ChE) for the selective and sensitive determination of total cholesterol using CV [141]. The prepared biosensor possessed sensitivity of 29.33 µA·mM^−1^·cm^−2^ with the K_m_^app^ of 0.64 mM, a linear range up to 300 mg·dL^−1^, and limit of detection 0.5 mg·dL^−1^. The biosensor was further used to detect cholesterol in real food samples margarine, butter, and fish oil with high sensitivity. Table 4 summarizes the analytical performance of different electrochemical methods that are based on np-Au electrodes prepared using different strategies for the detection of wide varieties of analytes.

### 6.3. Immunosensor

The development of sensitive and reliable diagnostic devices for various disease biomarkers is important for the early detection, management, and treatment of diseases [147]. Many disease biomarkers (such as antigens) are found as proteins and are specifically recognized from the biological fluids by a suitable antibody. In electrochemical immunosensors, any interaction between antigen and antibody produces electrical signals mostly in the form of electric current, changes in potential, or in resistance. When considering the presence of a very small concentration of the antigen, it is of utmost importance to amplify the electrochemical signal of the immunoreaction. In general, the signal can be amplified using enzymes linked to the antigen or antibody, which can catalyze the suitable substrate into the electroactive species after the immunoreaction [148]. However, due to good conductivity and the high surface area of np-Au, it can easily amplify the electrochemical signal with or without tagging the antibody. We have discussed earlier in the chapter that np-Au is a suitable support for the immobilization of enzymes or proteins due to its many important properties, including tunability, of the pore sizes for the convenient incorporation of proteins.

Electrochemical impedance spectroscopy (EIS), which is a powerful label-free electrochemical technique, is frequently used in the DNA sensor and enzymatic biosensor as a complementary technique for monitoring the multistep electrode modification. It is also one of the preferred methods in the immunosensing because of its sensitivity. Additionally, different important parameters can be recorded more explicitly, such as double layer charging, solution resistance, diffusion resistance and charge transfer resistance (R_ct_) [149]. The increase in R_ct_ value, observed as an increase in diameter of the semicircle in Nyquist plot, represents the increase in biomolecules density on the electrode surface. The calibration plot can be obtained by plotting change in R_ct_ versus the concentration of the target analyte. Using EIS, a limit of detection of as low as attomolar range has been reported for lectin-glycoprotein interaction on gold nanoparticles [150].

One of the early studies of impedance immunosensor based on np-Au uses a sandwich type assay, where the primary antibody was labeled with HRP [151]. When HRP catalyzes the hydrogen peroxide, the soluble compound 3,3′-diaminobenzidine tetrahydrochloride dehydrate converts to an insoluble product, which precipitates on the electrode surface, thus amplifying the resistance. Using this method, human immunoglobulin (IgG) was detected using goat-anti-human IgG antibodies with good linearity 0.011–11 ng·mL^−1^ and LOD of 0.009 ng·mL^−1^. In another work, a label-free impedance immunosensor was prepared based on np-Au (Rf = 19) to detect human serum albumin (HSA) using anti-HSA. The np-Au electrode was found to enhance the sensitivity up to 8.7 times over that of the flat electrode with the detection limit down to 10 fM [152]. With the better sensitivity of EIS comes the risk of false positive and negative results because of contaminations and nonspecific interactions.

Different other electrochemical techniques, such as CV, DPV, SWV, and CA have been used to prepare the np-Au-based immunosensor with or without the labeling of the antibody (Table 5). Using the labeling technique may enhance the electrochemical response, but labeling the primary antibody may also decrease its ability to interact with antigen. Label-free techniques are simple, easy to prepare, and the response can be obtained directly without the need of preparing sandwich type assay. Label-free immunosensor are designed based on a decrease in electrochemical signal (transfer of electrons) generated from redox species that are present in electrolyte solution due to the formation of antigen-antibody complex. The strategy has been applied to detect human serum chorionic gonadotropin (hCG) by taking advantages of high surface area of np-Au and graphene sheets using CV where hydroquinone was used as a redox species [153]. The prepared immunosensor has a wide linear range of 0.5–40.00 ng·mL^−1^, with the detection limit as low as 0.034 ng·mL^−1^. This label-free approach was further expanded by the same lab to detect cancer biomarker PSA with the linear range of signal reduction and LOD of 0.05–26 ng·mL^−1^ and 3 pg·mL^−1^, respectively [154]. Likewise, the antibiotic kanamycin was detected with the linear range of signal reduction and LOD of 0.02–14 ng·mL^−1^ and 6.31 pg·mL^−1^, respectively [156]. However, kanamycin was detected using square-wave voltammetry and the np-Au electrode was modified with Prussian blue, which works as electron transfer mediator.

The labeled immunosensors are commonly prepared as sandwich type assays. The primary antibody Ab1 can be immobilized on the np-Au surface to interact with target antigen Ag, which in turn, interacts with another primary antibody Ab2 tagged with enzymes or nanoparticles. In one study, the primary antibody Ab2 was labeled with sodium montmorillonites, aluminosilicate clay minerals, which were further modified by immobilizing thionine (TH) and horseradish peroxidase (HRP) to amplify the electrochemical signal to detect zeranol, a mycotoxin, using np-Au [155]. This signal amplified immunosensor can detect zeranol in the linear range of 0.01–12 ng·mL^−1^, with the LOD down to 3 pg·mL^−1^ using CV. Similarly, primary antibody Ab2 can be modified with enzymes and nanoparticles for the sensitive detection of other biomarkers using np-Au, such as carbohydrate antigen CA 72-4 and CA 15-3 [159,161]. Np-Au in the form of nanospheres has also been used as a nanoprobe, instead of an electrode, by loading it with glucose oxidase (GOx) and ferrocene (Fc) to enhance the electrochemical signal. As-prepared immunosensor was able to detect the CEA down to 0.45 pg·mL^−1^ [162]. Our laboratory has previously demonstrated the direct assay method for the detection of PSA and CEA on np-Au surface using SWV [157]. The assay was performed by immobilizing the alkaline phosphatase (ALP) conjugated antibody PSA or CEA on np-Au surface through the activated lipoic acid SAM. The SWV response was obtained from oxidation of p-aminophenol, the product of enzyme substrate p-aminophenylphosphate, near 0.1 V (vs. Ag/AgCl). The difference in peak current due to the oxidation of p-aminophenol increases with the increase in the concentration of PSA or CEA. Using this method, wide linear detection range of 1–30 ng·mL^−1^ and 0.2–10 ng·mL^−1^, with the limit of detection of 0.75 ng·mL^−1^ and 0.015 ng·mL^−1^, were obtained for PSA and CEA, respectively.

The sensitivity of immunosensors can be greatly improved by combining the high surface area of np-Au with luminophores (e.g., quantum dots and luminol) through the technique called electrochemiluminescence (ECL) [163]. By capturing CEA on the np-Au electrode via sandwich assay and labeling secondary antibody with CdTe quantum dots, a linear detection range of 0.05–200 ng·mL^−1^, and limit of detection 0.01 ng·mL^−1^ was obtained when potential is cycled between 0 and −0.2 V in 0.5 mol·L^−1^ H_2_SO_4_ [164]. A paper-based electrochemiluminescence immunodevice was prepared using nanoporous gold/chitosan as a sensor platform and graphene quantum dots functionalized Au@Pt core-shell nanoparticles as a signal label [165]. A sandwich type assay that was performed using this strategy to detect CEA shows a linear detection range from 1.0 pg·mL^−1^ to 10 ng·mL^−1^, with the detection limit of 0.6 pg·mL^−1^ [166].

## 7. Conclusions

Although self-supported np-Au is easy to prepare, using it as a biosensor is challenging because of its stability and electrical connection problems. This can be solved by fabricating solid-supported np-Au electrodes and employing electrochemical techniques for fabrication. Depending on the techniques that are used, np-Au having variable surface area and pore sizes can be prepared. Pore size, in particular, is of importance for the easy incorporation of biomolecules inside np-Au. Electrochemical techniques can fine-tune the pore sizes of np-Au. In this article, we discussed different electrochemical strategies to prepare and modify np-Au, along with the suitable electrochemical characterizing techniques. We further discussed the application of np-Au in electrochemical DNA sensor, enzymatic biosensor, and immunosensor.

Np-Au is of interest mainly because of its simple synthetic procedures, long-term stability, biocompatibility, and regenerability. These are the essential properties of the transducer of any chemical or biosensing devices for commercializing and pushing it to the state-of-the-art form. The scope of np-Au is broadening continuously because it can easily incorporate biomolecules on its surface without drastically changing its properties, and also because it can be easily modified by varieties of metal or metal oxides generating hybrid structures that are generating unique synergistic properties. The modified np-Au, when used as a transducer for chemical or biosensor, can highly enhance selectivity and sensitivity of the device to detect various toxic chemicals or disease biomarkers. Moreover, when metal or metal oxides-modified np-Au replaces biomolecule-modified np-Au in term of selectivity and sensitivity, it may be a huge advantage for the point-of-care devices. Unlike the metal and metal oxides, biomolecules tend to degrade faster with time, which may not be practical for the end users in a remote setting. Some researchers are already focusing in this area, but more works need to be done in this direction to obtain the suitable hybrid structures, depending on target analytes.

Because of the nanostructured features of the ligaments, np-Au is localized surface plasmon resonance active, and hence can be used as a transducer for a plasmonic-based optical biosensor. This makes np-Au a unique 3-D nanomaterial, which can be used as a transducer for both optical and electrochemical device. Future efforts should focus on using np-Au as a transducer that can work simultaneously in both electrochemical and optical sensors verifying the results of each other in real time.

The research on nanoporous gold and its use as a transducer for biosensing is continuously evolving. However, couples of practical challenges remain to be addressed. First, many innovative works have been limited to the laboratory scale. Second, due to either difficulty in obtaining the real sample or easy to finish the work on time, many experiments were performed in a model system. The results that were obtained from the model system can vary widely from that of the real sample, as the analyte in the real sample may be in the complex environment than that of model system. Nevertheless, with the revolution of the automation and the emerging of different nanomaterials including np-Au, the smart diagnostic tools in its best possible form is not far from realization.

## Figures and Tables

**Table 1:** Advantages and disadvantages of different np-Au fabrication techniques.

Method	Advantages	Disadvantages
Electrochemical etching of Au electrode	One-step process No need to prepare alloy beforehand No need of highly concentrated corrosive chemicals Low chances of impurity on surface	Difficult to control size of pores and ligaments Can be time consuming
Electrodeposition	One-step process No need to prepare alloy beforehand Stable and highly pure structure can be formed	Difficult to create thicker structure Difficult to control size of pores and ligaments
Dealloying (a) Chemical (b) Electrochemical	Easy and no need of instrumentation Large number of samples can be prepared at the same time in a batch Size of pores and ligament can be tuned easily Optimal for self-supported np-Au structures Better control over pores and ligaments size when an alloy is a thin layer No need of highly corrosive solvents	Use of corrosive solvents May contain impurities from less noble metals Once dealloyed (self-supported structures), difficult to use as a working electrode because of fragile nature and connection problem Time consuming if thicker and large number of electrodes have to be prepared Electrolyte gets contaminated after dealloying and may need to be changed after each dealloying

**Table 2:** Nanoporous gold modified DNA receptors for the detection of various analytes by electrochemical techniques.

Tech	Sensing Method	Analyte	Probe/Label	Linear Range	LOD	Ref.
CC	Hybridization	DNA	[Ru(NH_3_)_6_]^3+^	50–250 fM	5.6 fM	[97]
CC	Hybridization	DNA	AuNP/[Ru(NH_3_)_6_]^3+^	0.08–1600 fM	28 aM	[98]
CC	DNAzyme	Pb^2+^	[Ru(NH_3_)_6_]^3+^	0.05–100 nM	12 pM	[93]
CC	Aptasensing	Thrombin	AuNP/[Ru(NH_3_)_6_]^3+^	0.01–22 nM	30 fM	[90]
DPV	Hybridization	Hg^2+^	Ferrocene	0.01–5000 nM	3.6 pM	[78]
DPV	Aptasensing	Bisphenol A	-	0.1–100 nM	0.056 nM	[92]
DPV	Aptasensing	ATP	DABA	0.1–3000 µM	0.1 µM	[91]
DPV	Hybridization	DNA	Methylene blue	60–220 pM	6.7 pM	[101]
DPV	Hybridization	*E. coli*	Methylene blue	50–50000 cfu·µL^−1^	50 cfu·µL^−1^	[100]
DPASV	Hybridization	DNA	PbS-AuNP	0.9–70 fM	0.26 fM	[102]
SWV	Hybridization	DNA	[Fe(CN)_6_]^3−/4−^	10–200 nM	10 nM	[28]

CC: Chronocoulometry, SWV: square-wave voltammetry, DPV: differential pulse voltammetry, DABA: 3, 4-diaminobenzoic acid, DPASV: differential pulse anodic stripping voltammetry.

**Table 3:** Comparison of amperometric response of varieties of nanoporous gold (np-Au)-based glucose biosensors.

Electrode	Rf	Potential ^a^	Mediator	Linear (mM)	LOD (µM)	Sensitivity (µA·cm^−2^·mM^−1^)	Ref.
GOx-Chi/PB/np-Au/Ti	NA	−1.0 V ^b^	PB	0.1–2.0	2.5	177 ^c^	[119]
Naf/GOx/np-Au/GC	NA	0.4 V	-	1–18	196	0.049 ^c^	[30]
GOx/SAM/np-Au/GC	NA	−0.2 V ^b^	-	3–8	10	8.6	[110]
Naf/GOx/PB/np-Au/Cr/Si	40	0 V ^b^	PB	2–30	300	50	[120]
GOx/Os(bpy)_2_P/np-Au/SiO_2_	NA	NA	Os(bpy)_2_P	NA	2	75	[121]
GOx/PEDOT/np-Au/GC	NA	0.2 V	BQ	0.1–15	10	7.3	[122]
GOx/np-Au/Au/Si	36	NA	-	0.1–0.5	73	21.14	[123]
GOx/GA/CA/np-Au/GC	7	0.2 V 0.3 V	BQ FCA	1–10 1–10	NA NA	3.53 1.35	[124]
GOx/DTDPA/np-Au/GC	8	0.2 V	BQ	1–10	NA	2.187	[9]
GOx/np-Au/GC	8	0.3 V	-	0.05–10	1.02	12.1	[125]

^a^ vs. SCE, ^b^ vs. Ag/AgCl, ^c^ µA·mM^−1^, Rf: roughness factor of np-Au, Chi: chitosan, GC: glassy carbon electrode, Naf: Nafion, PB: Prussian blue (ferric hexacyanoferrate), DET: direct electron transfer, BQ: p-benzoquinone, FCA: ferrocenecarboxylic acid, GA: glutaraldehyde, CA: cysteamine, DTDPA: 3,3′-Dithiodipropionic acid, PEDOT: poly(3,4-ethylenedioxythiophene), Os(bpy)_2_P: [Os(2,2′-bipyridine)_2_(polyvinylimidazole)Cl]^+/2+^.

**Table 4:** Detection of varieties of analytes using enzymes immobilized on nanoporous gold (np-Au) surface by different electrochemical techniques.

Tech	Electrode	Analyte	Linear (mM)	LOD (µM)	Sensitivity (µA·cm^−2^·mM^−1^)	Ref.
CA	FDH/ND/np-Au/glass	Fructose	0.05–0.3	1.2	3.7	[140]
	Nafion/ADH/np-Au/GC	Alcohol	1.0–8.0	120	0.19 ^a^	[30]
	Cyt c/np-Au/ITO	H_2_O_2_	0.010–12	6.3	2.8	[142]
	HRP/np-Au/Au	H_2_O_2_	0.010–0.380	2.6	21	[106]
CV	ChOx+ChE+HRP/np-Au/Ti	Cholesterol	0.97–7.8	12.9	29.33	[141]
	Lipase/np-Au/GC	Tributyrin	1.65–8.27	88.6	0.009	[143]
	AChE/MWCNT/Ci/np-Au/Au	Malathion	0.003–0.150	0.0015	NA	[144]
DPV	HRP/np-Au/GC	Catechol	7–150	0.66	31.8	[145]
	HRP/np-Au/GC	Sulfide	0.1–40	0.027	1720	[146]

FDH: fructose dehydrogenase, ND: 2-carboxy-6-naphtoyl diazonium, ADH: alcohol dehydrogenase, GC: glassy carbon electrode, HRP: horseradish peroxidase, GOx: glucose oxidase, Cyt. C: cytochrome c, ChOx: cholesterol oxidase, ChE: cholesterol esterase, AChE: acetylcholinesterase, Ci: cysteamine, ^a^: µA·mM^−1^.

**Table 5:** Nanoporous gold (np-Au)-based immunosensors for the detection of antigens using different electrochemical techniques.

Tech	Ab/Electrode	Antigen	Label/Probe	Linear	LOD	Ref.
EIS	Ab1/np-Au/GC	human IgG	Ab2-HRP	0.011–11 ng·mL^−1^	0.009 ng·mL^−1^	[151]
EIS	Ab/11-MUA/np-Au/Au	HSA	Label-free	0.010–10,000 pM	10 fM	[152]
CV	Ab/np-Au/GS/GC	hCG	Label-free	0.5–40.00 ng·mL^−1^	0.034 ng·mL^−1^	[153]
CV	Ab/np-Au/GC	PSA	Label-free	0.05–26 ng·mL^−1^	3 pg·mL^−1^	[154]
CV	Ab1/np-Au/GC	zeranol	Ab2/HRP/TH/SM	0.01–12 ng·mL^−1^	3 pg·mL^−1^	[155]
SWV	Ab1/np-Au/PB-C/GS /GC	kanamycin	Label-free	0.02–14 ng·mL^−1^	6.31 pg·mL^−1^	[156]
SWV	Ab1-ALP/LA/np-Au	PSA CEA	ALP	1–30 ng·mL^−1^ 0.2–10 ng·mL^−1^	0.75 ng·mL^−1^ 0.015 ng·mL^−1^	[157]
DPV	Ab1/DTSP/np-Au/GC	HBsAg	Ab2-HRP/AuNPs	0.01–1.0 ng·mL^−1^	2.3 pg·mL^−1^	[158]
DPV	Ab1/TH/np-Au/GS/GC	CA 15-3	Ab2/HRP@Lip	2 × 10^−5^–40 U·mL^−1^	2 × 10^−6^ U·mL^−1^	[159]
DPV	Ab1/AuNP/TH/np-Au/GC	CEA	Label-free	0.01–100 ng·mL^−1^	3 pg·mL^−1^	[160]
CA	Ab1/np-Au/GC	CA 72-4	PANI/Au AMNPs	2–200 U·mL^−1^	0.10 U·mL^−1^	[161]

Ab: antibody, npAu: nanoporous gold, GC: glassy carbon electrode, HRP: horseradish peroxidase, 11-MUA: 11-mercaptoundecanoic acid, HSA: human serum albumin, GS: graphene sheet, hCG: human serum chorionic gonadotropin, PSA: prostate specific antigen, TH: thionine, SM: sodium montmorillonites, PB-C: Prussian blue-chitosan, ALP: alkaline phosphatase, LA: lipoic acid, DTSP: 3,3′-Dithiodipropionic acid di(*N*-succinimidyl ester), HBsAg: hepatitis B surface antigen, CA: cancer antigen, Lip: liposome, AuNP: gold nanoparticles, CEA: carcinoembryonic antigen, PANI/Au AMNPs: polyaniline–Au asymmetric multicomponent nanoparticles.
